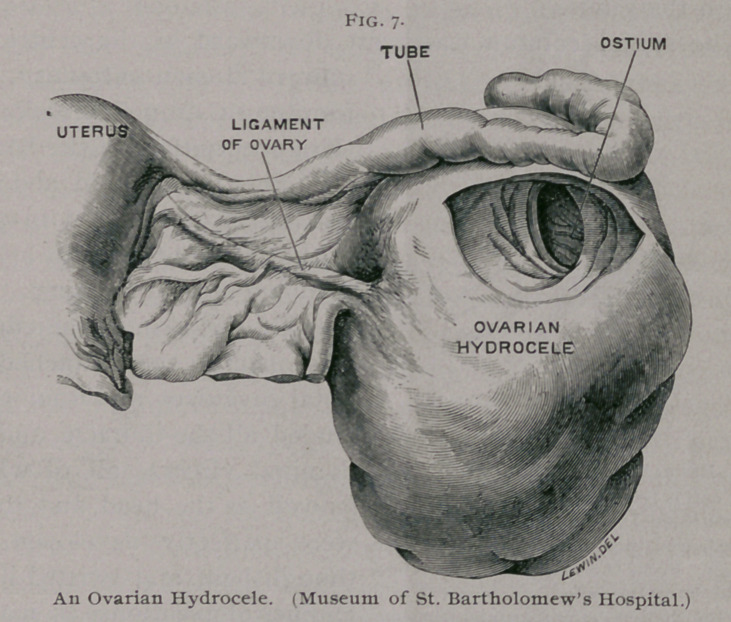# Comparative Pathology1Abstract of an Introductory Lecture to a Course of Comparative Pathology, delivered at the Royal Veterinary College, London, October, 1890.

**Published:** 1891-01

**Authors:** J. Bland Sutton


### THE JOURNAL
OF
### COMPARATIVE MEDICINE AND VETERINARY ARCHIVES.

VOL. XII. JANUARY, 1891. NO. 1.

### COMPARATIVE PATHOLOGY.[1]

By J. BLAND SUTTON, F. R. C. S.

GENTLEMEN—With commendable judgment your Principal decided that it is desirable to institute a Chair of Comparative Pathology. This is a decision of no little importance, for it is the first time that this subject has been awarded so dignified a position in a college, veterinary or otherwise, in Great Britain.

You may well feel that the time at your disposal as students of the Veterinary Art is very limited, and this new impost may not be agreeable to some, but I will promise to make the subject interesting as far as lies in my power. For my own part, I look forward to our meetings with great pleasure, and feel sure that many of you will be able and willing to assist me in securing pathological specimens in order to demonstrate some of the principles which go to make up the Science of Pathology.

At the outset let me say candidly, that I shall tell you little which will be of use in the practice of the Veterinary Art, for Comparative Pathology bears much the same relation to it as the Science of Botany bears to Agriculture. Nevertheless, I can venture to predict that you will be none the worse for a knowledge of the facts and theories which belong to Comparative Pathology, and its study will often relieve the tedium of routine work and lead to broader conceptions of disease in general. Much of the mysticism formerly current in the profession of medicine (much

[1] Abstract of an Introductory Lecture to a Course of Comparative Pathology, delivered at the Royal Veterinary College, London, October, 1890.

yet persists) was due to ignorance of the fundamental principles of Pathology. Since Pathology has been more thoroughly studied medicine has become more and more of a rational art, although she is still fettered by traditionalism and the want of free thought among her followers.

Comparative Pathology is important to those who study the diseases of the horse as well as to those who study the diseases of mankind. The horse and man are exceedingly specialized mammals. Hippo-pathology is as special a study as Human-pathology, but Comparative Pathology is a department of Biology concerned in collecting facts connected with abnormal deviations in all living things; arranging the facts in groups in order to detect laws which underlie the origin of those aberrations of structure and function to which the term Disease is applied. It is therefore a wide subject of study, so wide, indeed so limitless, that to-day we shall merely consider a few examples which will serve to show the valuable aid Comparative Pathology lends to Special Pathology; how it throws light in unexpected directions and renders many obscure conditions intelligible without the amusing explanations of Teleology.

Few conditions illustrate more forcibly the advantage of studying Pathology in a comprehensive manner than the peculiar condition of the ovary and tube, often called *Tubo-ovarian cyst*, but which I shall prefer to speak of as

### OVARIAN HYDROCELE.

True ovarian cysts (cysts arising in the egg-bearing portion of the ovary) do not burrow between the layers of the mesometrium (suspensory ligament of the uterus), consequently they do not involve the Fallopian tube. The tube may, however, adhere to the walls of the cyst in consequence of inflammation, and its abdominal ostium may become closed by adhesion of its fringes, but the tube never communicates with the interior of an ovarian cyst.

There is a rare form of cyst which I call ovarian hydrocele, in which the ampulla of the tube is dilated, tortuous and communicates by a large orifice with the interior of a cavity filled with fluid occupying the normal position of the ovary. The cyst and tube may be so intimately connected that it is impossible to determine where the tube ends, and the cyst begins. A tubo-ovarian hydrocele has been aptly compared to a retort with a convoluted delivery tube.

These hydroceles are not confined to the human female, for Schneidemühl[1] has described and figured a typical specimen which he obtained from a mare. He says the ovary was transformed into a pear-shaped bladder. It was 26 cm. long and 18 cm. wide, and contained fluid of a clear golden yellow color. The lumen of the tube was directly continuous with the cavity of the cyst.

FIG. 1.

An Ovarian Hydrocele (human).

The ovary in many mammals lies in recess of mesometrium termed the ovarian pouch. In the majority of mammals the pouch is very shallow, in others it is deep, and in a few it forms an investment for the ovary as complete as the tunica vaginalis which invests the testes, but with this difference, the Fallopian tube communicates with the interior of the pouch.

The mode in which the ovarian pouch is formed has recently been investigated by McArthur Robinson.[2] The mammalian uterus, whether bicornuate or single, is attached to the abdominal walls by means of a peritoneal fold; the mesometrium, broad or suspensory ligament. In some mammals, with bicornuate uteri,

[1] Deutsche Zeitschrift für Thiermedecin. Bd. ix, S. 279, Beitrag zur Casuistik der Tubo ovarialcysten beim Pferde.

[2] Journal of Anatomy and Physiology, 1887. On the position and peritoneal relations of the mammalian ovary.

each ovary is immediately posterior to the kidney and is attached to the mesial layer of the corresponding mesometrium. In mammals in which the ovaries recede to the pelvis, the ovaries are still found attached to that layer of the mesometrium which was mesial, but now becomes dorsal. As a rule, the ovary is in shape a laterally compressed ovoid. One border is attached to the mesometrium, the other is free. Each extremity is connected with a ligament; one, the *ovarian ligament* is connected with the uterine cornu, the other is called the diaphragmatic ligament, and is either lost in the mesometrium (broad ligament) or, under certain conditions, extends to the kidney or diaphragm. These bands give rise to reduplications of the peritoneum and cause a shallow depression in that portion lying between the ovary and tube, conveniently termed the mesosalpinx. This depression may become so pronounced as to constitute a distinct sac.

FIG. 2

Ovarian Hydrocele in a Mare (after Schneidemühl).

FIG. 3.

Transverse Section of the Ovary and Ovarian Pouch of a Mouse (after Robinson).

The Fallopian tube lies between the layers of the mesometrium, and in a few occupies the free edge of this structure; in many a fringe of this peritoneal fold extends beyond the tube. The abdominal ostium of the tube is funnel-shaped, but compressed so as to form an elongated slit; one extremity of the slit is attached to the ovary, the other is free. The orifice of the tube is parallel with the ovary and on the margin of the ovarian pouch.

As one end of the slit-like ostium is fixed, it necessarily follows that if the tube lengthen it must push forward between the layers of the mesosalpinx and become tortuous. Increase in the length of the tube is accompanied by growth of the mesosalpinx and serves to deepen the pouch which tends to invest the ovary. This condition is seen in the baboon (*cynocephalus porcarius*. Fig. 4). The pouch becomes more complete in the porcupine in consequence of adhesion of the free edge of the mesometrium at the uterine end of the tube to the fold formed by the ovarian ligament, reducing the orifice of the sac to a mere slit scarcely exceeding the length of the ovary. (Fig. 5.)

In a few mammals (badger, raccoon), the edge of the slit adheres to the attached border of the ovary, leaving merely a minute orifice near the uterine end of the ovary. In these mammals the pouch tightly invests the ovary. In the mouse and rat Robinson has shown that even this orifice is wanting, so that the ovary is shut off from the peritoneal cavity, and the abdominal ostium of the tube opens in the pouch.

FIG. 4.

Ovarian Pouch of a Baboon. (After Robinson.)

Ovarian pouches have long been known with the exception of the complete form described by Robinson, and we are entirely indebted to him for working out the method by which the pouch is formed. From what we know of the tendency of the peritoneal pouch of the testes to become the seat of accumulation of fluid (hydrocele), it occurred to me to look for similar hydroceles of the ovarian pouch, for it was reasonable to believe that these pouches would occasionally be distended with fluid.

Fig. 5.

Ovarian Pouch of a Porcupine. (After Robinson.)

The Museum of University College contains such a specimen, supposed to be an example of bilateral ovarian cysts in a guinea-pig.

The parts are sketched below. (Fig. 6.) The Fallopian tubes, much elongated and convoluted, pass round the outside of the almost globular translucent cysts. Their terminal segments are somewhat dilated, but the abdominal orifices are obstructed. The orifice of the tube is indicated by a circular depression on the wall of the cyst, and a few low ridges radiate from it.

Fig. 6. Ovarian Hydroceles in a Guinea-Pig.

On the wall of each pouch is the cystic remnant of the ovary, which bears precisely the same relation to the cyst that the testis bears to a hydrocele of the tunica vaginalis. This specimen from the guinea-pig is peculiarly interesting, because the ovarian pouch of this rodent resembles that of the porcupine, so that abnormal union of the edges of the slit (see Fig. 5) must have taken place to form a complete capsule.

With these facts in my mind, I re-examined an excellent specimen, described as a tubo-ovarian cyst by Dr. Griffith, which is preserved in the Museum of St. Bartholomew's Hospital. The parts consist of the uterus with the broad ligaments (mesometria), removed after death from a woman 27 years of age. The right side of the uterus, with the mesometrium, are sketched in Fig. 7. The right tube measures 23 cm. in length. The uterine segment is of normal size, but the outer third greatly distended and convoluted opens by a large circular orifice (5 cm. in diameter) into the cavity of a very thin-walled unilocular cyst measuring 13 cm. by 9 cm., which projects from the posterior surface of the right broad ligament. A few low ridges are seen at the spot where the tube and pouch become continuous. At the base of the cyst there is a stratum of ovarian tissue in relation with the ovarian

FIG. 7.

An Ovarian Hydrocele. (Museum of St. Bartholomew's Hospital.)

ligament. The cyst contained a thin, almost colorless fluid. My observations lead me to believe that this cyst is a distended ovarian pouch of the same nature as those in the guinea-pig, and the pressure of the fluid induced atrophy and flattening of the ovary.

Thus the anatomical evidence indicates clearly enough that these cysts are similar to hydroceles occurring in relation with the tunica vaginalis testis and, in course of time, exercise the same influence upon the ovary as upon the testis and induce atrophy from pressure.

## Figures and Tables

**Fig. 1. f1:**
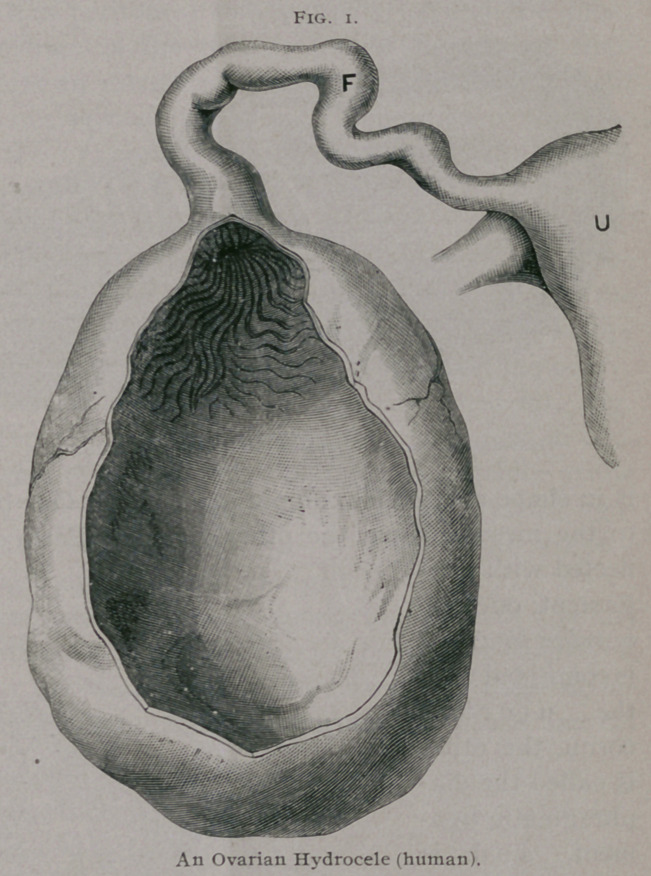


**Fig. 2 f2:**
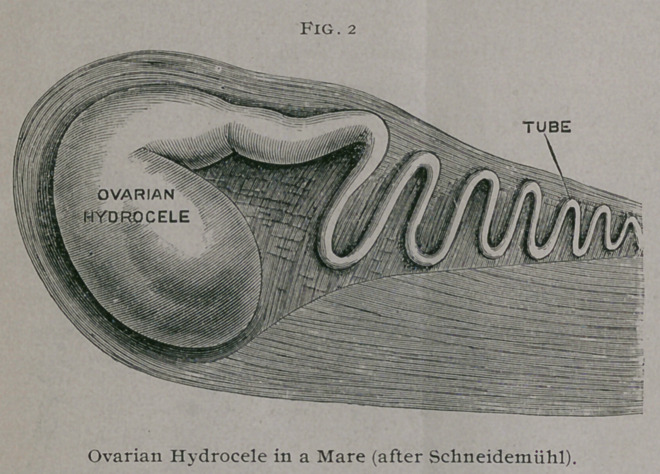


**Fig. 3. f3:**
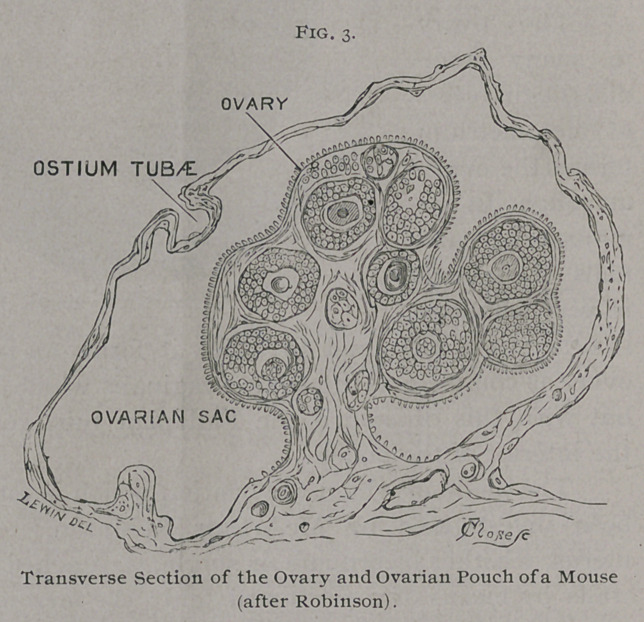


**Fig. 4. f4:**
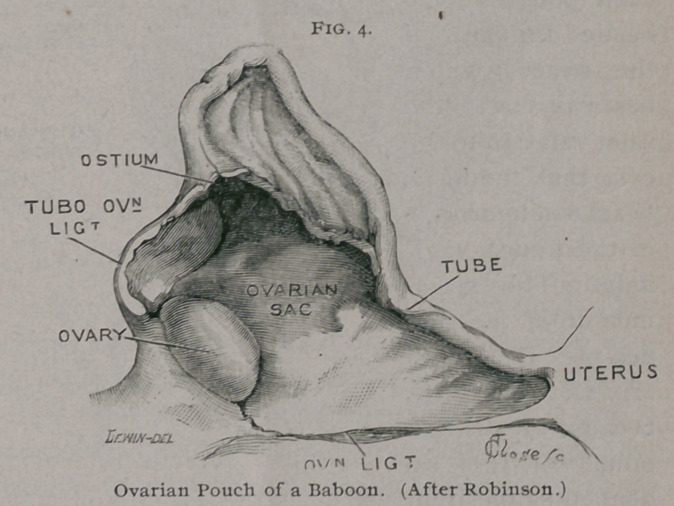


**Fig. 5. f5:**
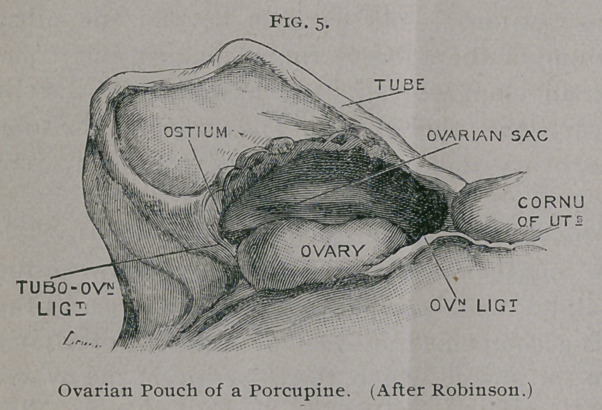


**Fig. 6. f6:**
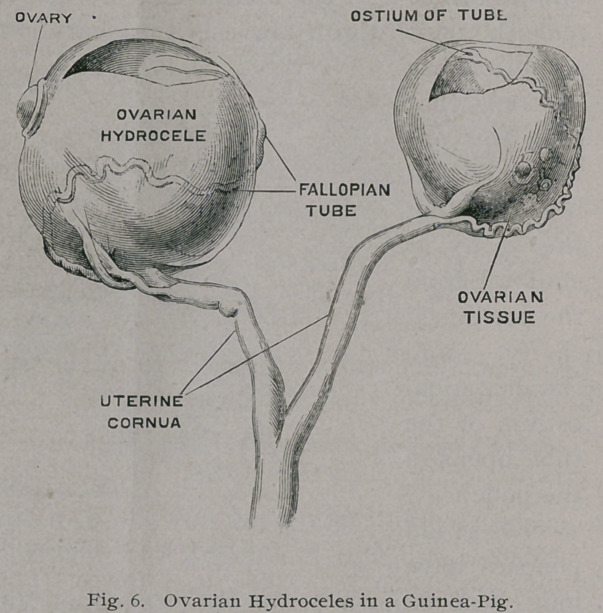


**Fig. 7. f7:**